# Enhancing resilience in transnational E-commerce supply chains: Critical factors, perspectives and strategic action plan

**DOI:** 10.1016/j.heliyon.2024.e31274

**Published:** 2024-05-16

**Authors:** Dewan Hafiz Nabil, Md Al Amin, Roberto Baldacci

**Affiliations:** a Department of Industrial Engineering and Management, Khulna University of Engineering & Technology, KUET-9203, Bangladesh; b Division of Engineering Management and Decision Sciences, College of Science and Engineering, Hamad Bin Khalifa University, Doha-34110, Qatar

**Keywords:** Transnational e-commerce, SC resilience, ENTROPY-SAW-ISM model, SWOT analysis

## Abstract

This study develops a hybrid model to investigate the factors affecting transnational e-commerce supply chain resilience (TNSCRE) by integrating the Entropy Weight Method (EWM), Simple Additive Weighting (SAW), and Interpretive Structural Modeling (ISM). The study identifies 36 critical factors categorized under supply chain adaptability, supply chain efficiency, and supply chain evolution, and five criteria are used to rank these factors. The EWM is used to calculate the relative weights of the criteria, and the SAW method is used to rank the factors based on their weighted scores. The ISM is then used to evaluate the interrelationships among the key factors. The research highlights the significance of several factors, such as the speed of supply chain disruption recovery, interactive collaboration, and response time to supply chain disruption. Sensitivity analysis was performed to assess the robustness of the findings. Finally, a SWOT analysis is conducted to develop a strategic action plan for addressing these significant factors. The study provides a comprehensive understanding of the factors that impact TNSCRE from the perspective of multiple stakeholders. The findings can help e-commerce business owners improve their existing supply chain resilience and achieve sustainable growth in the context of globalization.

## 1 Introduction

As the global economy has become more integrated, Transnational electronic commerce (TEC), also known as cross-border e-commerce, has emerged as a new driver of international trade growth, particularly in Bangladesh [1]. TEC represents a unique form of transnational trade characterized by the electronic exchange of goods and services across borders. Unlike traditional international trade, TEC leverages online marketplaces and digital platforms to connect buyers and sellers globally. Transactions occur electronically, often bypassing physical stores and intermediaries. TEC encompasses a diverse range of products, both physical and digital. While physical goods like clothing, electronics, and household items form a significant portion of TEC, it also includes digital products like software, e-books, and music. Notably, the specific supply chain requirements for TEC products differ depending on their characteristics. Physical goods necessitate traditional logistics management, including packaging, warehousing, and international shipping. Perishable goods like fruits and vegetables require specialized cold-chain logistics to maintain freshness during transport. In contrast, digital products leverage digital delivery infrastructure, eliminating the need for physical transportation. This very diversity in product types and their corresponding supply chains is crucial when analyzing factors impacting Transnational Electronic Commerce Supply Chain Resilience (TNSCRE). Traditional international trade often focuses on bulk commodities or manufactured goods and may have standardized logistics processes. However, TEC demands adaptability and flexibility to accommodate the varied needs of different product categories within a single business model [2].

Despite its many advantages, such as enabling Bangladeshi businesses engaged in electronic commerce to participate in the global trade and supply chain, the sector is more exposed to risks posed by the external system, like theft, natural disasters, political unrest, financial instability, and lawsuits. These dangers increase TEC's vulnerability and potential for supply chain disruption. As a result, supply chain resilience (SCRE) is a strategic tool for guaranteeing the stability and safety of TNSCRE and is essential for Transnational e-commerce business owners to keep their competitive advantages and stay ahead in the global market [3]. However, when considering the complicated structure underlying transnational e-commerce supply chains, it is necessary to consider what factors will impact SCRE, how they will affect it, and the relationships between them. Additionally, transnational e-commerce enterprises should focus on the steps they can take to optimize their supply chains and enhance their SCRE. Exploring the factors that influence TNSCRE, considering the variety of products being traded, is an important task in this field and will aid in resolving these issues.

Academic researchers, both domestically and internationally, have given significant attention to analyzing the factors that impact supply chain resilience, resulting in a range of valuable findings from various perspectives. These include research on the correlation between organizational innovation and supply chain resilience [4], Agility and flexibility of resilient supply chain [5] and Redundancy of SCRE [6]. Despite the progress made in understanding the impact factors of SCRE, some areas still need further investigation. The initial point to note is that most studies concentrate on examining a single or limited number of factors, with a shortage of systematic research in the area. The lack of research findings on the impact factors of TNSCRE highlights the need for establishing a structured system of such factors. Additionally, the examination of the relationship between top impact factors is limited despite its being beneficial for determining the significant factors that drive TNSCRE. Consequently, it is essential to set up a logical hierarchy of TNSCRE's impact factors in the field. Providing strategic action plans to help business owners address the top critical factors is also significant for achieving SCRE.

This study aims to address the existing research gaps by creating a comprehensive set of influencing factors of TNSCRE, ranking them based on importance, constructing a hierarchical arrangement of these factors and finally providing a strategic action plan to address the critical factors. The study of the TNSCRE's influencing factors is hypothesized as a multi-criteria decision-making (MCDM) problem in which conflicting factors need to be evaluated and selected. MCDM is a crucial aspect of analytical decision theory and is often utilized to analyze the impact of various factors [7]. The next phases of the study will address the following research questions in order to achieve the research objectives.

1. What are the most critical factors influencing Transnational e-commerce supply chain resilience?
2. How are these factors ranked based on their importance in terms of achieving supply chain resilience?
3. How do the factors interrelate with each other in the context of Transnational e-commerce supply chain resilience?
4. How can organizations effectively formulate strategic plans to enhance their supply chain resilience in the context of globalization based on the identified factors and findings from the study?

This research set criteria weights based on the Entropy Weight Method (EWM) to rank the identified factors. Criteria weights are typically assigned subjectively using methods such as the Delphi method, analytic hierarchy process (AHP), and expert survey method. However, these methods can be biased and lead to inconsistent criteria weights. On the other hand, EWM eliminates human bias by determining criteria weights based on the inherent information of the criteria [8]. The EWM has been used in many supply chain research fields, such as evaluating blockchain-based healthcare supply chains [9], selecting suppliers in sustainable supply chains [10] and managing ripple effect environmental risks in supply chains [11]. In MCDM, Simple Additive Weighting (SAW) is a computationally efficient method, making it well-suited for ranking 36 factors in our study [12]. Many experts have used SAW and ISM to analyze and rank factors related to Supply chain analysis (SCA). For example, Güler et al. [13] used SAW to assess the success factors for AI application in supply chain management, Jaberidoost et al. [14] used SAW to assess pharmaceutical supply chain risk factors in Iran, Shweta et al. [15] used ISM to explore the issues of Indian generic medicine supply chain, Badhotiya et al. [16] used ISM to identify and analyze the key factors that influence the resilience of supply chains in Indian manufacturing firms during the COVID-19 pandemic, and Negri et al. [17] used SAW and ISM to develop a performance measurement framework for supply chain sustainability and resilience. SAW is also easy to understand and interpret, which can help build consensus and make informed decisions among stakeholders of Transnational e-commerce businesses [18]. However, SAW does not consider the interactions between factors. To address this limitation, we integrated SAW with Interpretive Structural Modeling (ISM), which excels at uncovering the logical hierarchy of factors. After all aspects were taken into account, this study develops a comprehensive system of the TNSCRE's influencing factors by combining the core theory of SCRE and a literature review. Reliability analysis is performed using SPSS to assess the internal consistency of the factors. The EWM and SAW methods are then combined to rank the TNSCRE influencing factors. The ISM method is also used to create a multi-level hierarchy of TNSCRE's influential factors. Finally, a SWOT analysis is conducted for strategic planning.

The novelty of this research lies in its innovative approach to the emerging field of TNSCRE. Unlike previous studies that often concentrated on isolated aspects of supply network efficiency, this research boldly incorporates the core SCRE theory to identify critical factors impacting TNSCRE, providing a more comprehensive perspective. Additionally, the introduction of a hybrid model that integrates the EWM, SAW, and ISM represents a novel and sophisticated analytical framework, offering deeper insights into TNSCRE dynamics. Furthermore, the utilization of the ISM technique to construct a hierarchical structure of influencing factors adds an innovative dimension to the research, revealing the complex interplay among contextual variables and highlighting the most pivotal elements for TNSCRE improvement. The inclusion of a strategic action plan through SWOT analysis further underscores the research's innovation, offering practical guidance for business owners. Moreover, the focus on emerging Bangladeshi e-commerce businesses introduces a unique and understudied perspective, making this research a trailblazing contribution to the TNSCRE field. Overall, its innovative methodology, holistic insights, and applicability make this research a standout in the domain of TNSCRE.

Following that, a systematic flow structure is followed as mentioned. A review of the TNSCRE-influencing factors and the development of a thorough system of TNSCRE-influencing factors are provided in Section 2. The analytical process and findings are presented in Section 3 using the combined AHP-SAW-ISM methodology. The proper applications of the MCDM approach in this case are examined in Section 4 and numerical illustrations are highlighted in Section 5. The results, discussion and interpretation of transnational electronic commerce enterprises are presented in Section 6. Section 7 concludes by highlighting the contributions and lacking's of the study and proposing potential areas for future research.

## 2 Literature review

### 2.1 Factors identification

The SCRE analysis explores a system's timeframe to recover after an interruption has had an effect on it [19]. According to this analysis, these influencing factors are crucial components that facilitate supply chain resilience [20]. According to the SCRE hypothesis, numerous factors influence SCRE, including the capacity of the supply chain for efficiency, adaptation, and evolution. According to this study, the factors affecting transnational electronic commerce are grouped into three (3) primary categories: Supply Chain Adaptability, Supply Chain efficiency, and supply chain evolution.

#### 2.1.1 Supply chain adaptability

Supply chain adaptability, as defined by Ref. [21], denotes a supply chain's capacity to undergo multiple transitions and recover effectively following disruptions. Based on prior research, this study splits this concept into four categories: Supply chain flexibility, redundancy, Adaptive management, and Risk reduction practice. Supply chain flexibility includes a supply network's capacity to react efficiently to disruptions [22]. This can involve appropriate measures by management, flexible resources, and being acquainted with international e-commerce supply chain management [23]. In addition, Pettit et al. [24] advocate for greater flexibility in research and development, sourcing, transportation, and supply chain structure flexibility [25]. Supply chain redundancy is the diversity of components in an organization that performs the same job, enhancing the supply chain's adaptability [22]. For example, this would involve a multi-vendor approach to procurement, a variety of solutions for foreign logistics and storage, and a safety stock for international e-commerce [5]. Hendricks et al. [26], in particular, mention the availability of multiple modes of international transport and storage capacity [26]. The term “adaptive management” describes a system's ability to reconfigure itself in response to interruptions and changing conditions [27]. This might include the ability to address the potential to sustain short-term disruption [28]. For example, Jia et al. [3] discuss how adaptive management involves readiness to handle pre-disruption. By experimenting with interventions and learning from the system, this approach enables managers to cope with long-term disruption [29]. Risk reduction practices are established through a series of efforts and entail a supply chain's proactive attempt to manage disruptive occurrences [4]. This may entail a Collaborative risk reduction team, a fallback plan, and create an environment where partners can work together [30]. Rungtusanatham et al. [31] further advise creating cultural standards for cooperation and a continuous contingency plan.

#### 2.1.2 Supply chain efficiency

Supply chain efficiency focuses on determining how quickly the supply chain can respond [32]. To get a more concise idea, supply chain efficiency capabilities are categorized by four segments: crisis management, balancing supply and demand, Business improvement strategy, and agility of supply chain. It does this by considering the research of relevant researchers. Crisis management is critical to improving supply chain efficiency, including developing a supply chain contingency strategy to reconfigure any disruptions [33]. The supply chain contingency plan reconfiguration [34], contingency strategy for supply chain disruption recovery [35], and contingency plans for resource reallocation [25] can all be added to this to improve it further. Various emergency strategies, including the recruitment and coordination of overseas distributors, the recovery of thresholds, and the use of inventory storage techniques such as sub-contracted warehouses or overseas warehouses, have been put up by various researchers [36]. The ability to match the supply with demand is yet another indispensable tool for enhancing the supply chain's effectiveness, including tactics like deferred market segmentation and delayed use [23]. Waiting to invest in things until the very last minute to save money helps to lower risks. It can be made more expensive by designing generic products in response to customer needs, tailoring general products [37], and employing delay strategies to satisfy customer expectations [38]. Strategies for business improvement can be used to mitigate supply vulnerabilities [21]. Supply risks can be extremely tough to estimate and, should they materialize, have a disastrous impact on the firm. By analyzing the effect of key suppliers, evaluating the degree of risk that key suppliers pose [39], and identifying warning signs of supply chain hazards, this can be expanded further by taking into account the geographic location and national policies of suppliers and manufacturers or calculating the possible effects of losing raw material suppliers by analyzing risks [1]. The concept of “supply chain agility” entails the organization's ability to tackle the fluctuations in demand and supply [40]. This can be strengthened by taking into account variables like time required to respond to an interruption of the supply chain [32], rate of recovery [21], and Awareness about the supply chain's major links [41]. Globally, this includes the speed with which the Transnational e-commerce market responds to changes in demand, the identification of vulnerable suppliers, the environment of global electronic-commerce, and major resource requirements, which are the most obvious attributes of the core links in SCRE.

#### 2.1.3 Supply chain evolution

The capacity of the supply chain to change is emphasized by modernizing and improving the SC recovery and reconfiguration speed [21]. Based on the findings of relevant experts, this research work has sub-grouped to four sub-factors: knowledge management, Innovation capacity, collaboration among partners, and information sharing between stakeholders. By enabling the recording and use of vital information to address future changes, social knowledge management plays a critical part in enhancing the evolution of the supply network [42]. This can include utilizing risk experience [43,44], Earnestly gather social relationship assets and Utilizing managing risk experience. For example, Song et al. [44] emphasize the significance of upholding social relationships through time, energy, and resources and propose that long-term collaborations can result in important knowledge and asset management. The ability to innovate is yet another critical enabler for enhancing supply chain performance. A company's capacity to make changes is enhanced by effective learning, especially when it comes to handling confusing issues in dynamic systems [45]. This can include Individual innovation potentiality to adapt to risks [46] as well as combined innovation ability to adapt to risks [47]. Employees should be capable of self-directed learning, application, method discovery, and experience summarization, according to Patriarca et al. [48], With regard to Transnational e-commerce supply chain partners, Hosseini et al. [47] advise using organizational innovation capability to adapt risks [49]. The enhancement of supply chain evolution depends heavily on partners providing information, especially when there is trust built on goodwill [50]. Trust makes social interactions dependable, develops community, encourages cooperation, and lowers risk. This can include establishing integrity practices among Transnational e-commerce partners, maintaining agreements with multinational e-commerce partners in information sharing, and developing a positive corporate reputation for fairness [51]. Transnational e-commerce businesses should Create a positive business image for fairness in their respective industries [52,53]. In order to tackle inter-organizational issues that cannot be resolved individually, participants must be able to work effectively with other businesses through collaboration [24]. Exchange of knowledge between multinational e-commerce partners, Collaborative planning, and Interactive collaboration among multinational e-commerce partners are a few examples of this [36] and other Transnational e-commerce partner activities. Transnational e-commerce supply chain partners should prioritize knowledge generation and exchange [54]. Transnational e-commerce supply chain partners may support one another when there are disruptions to increase cohesion.

Table 1 summarizes the influencing factors of TNSCRE, layered relationships, and classification of transnational e-commerce SCRE based on the literature study and integrated with the features of transnational e-commerce supply chains. In conclusion, this study develops a TNSCRE influencing factor system that consists of 36 tertiary-level influencing factors in addition to 3 basic influencing factors and 12 supplementary factors.

**Table 1:** Influencing factors of TNSCRE.

Primary	Secondary	Third Level	Index	Reference
Supply Chain Adaptability	Supply chain flexibility	Acquainted with international electronic-commerce SC management	A1	[1,23]
		Resource flexibility of multinational e-commerce companies	A2	[24]
		SC structure flexibility for multinational e-commerce	A3	[55,25]
	Supply chain redundancy	Safety stock inventory for multinational e-commerce	A4	[5]
		Multiple vendor procurement	A5	[26]
		Availability of multiple modes of Transnational transport and warehouse capacity	A6	[26]
	Adaptive management	Capability to cope with long-term disruption.	A7	[56]
		Readiness to handle pre-disruption	A8	[3]
		Potential to sustain short-term disruption	A9	[57]
	Risk reduction practice	Create an environment where partners can work together	A10	[30]
		Collaborative risk reduction team.	A11	[30]
		Mutual contingency plan among partners	A12	[31]
Supply Chain efficiency	Supply chain agility	Speed of Recovery from disruption	A13	[21]
		Awareness of the supply chain's major links	A14	[41]
		Time required to respond to supply chain disruption	A15	[32]
	Capacity for supply and demand	Designing generic product	A16	[37]
		Customize generic products depending on the specifications	A17	[38]
		Intelligently utilize delay to meet client needs	A18	[23]
	Business improvement strategy	Evaluate the degree of risk that key suppliers pose.	A19	[39]
		Detect warning signals of supply chain risk	A20	[21]
		Assess the impact of key suppliers	A21	[39]
	Crisis strategy	Supply chain contingency strategy reconfiguration.	A22	[20,58]
		Assets reallocation contingency strategy	A23	[25]
		Supply chain disruption recovery contingency strategy	A24	[35]
Supply chain Evolution	Knowledge management	Acquired experience in response to risks	A25	[59]
		Utilization of managing risk experience	A26	[44]
		Earnestly gather social relationship assets	A27	[44]
	Innovation capacity	Individual innovation potentiality to adapt risks	A28	[46]
		Organizational innovation capability to adapt risks	A29	[47]
		Combined innovation ability to adapt risks	A30	[49]
	Collaboration among e-commerce supply chain partners	Exchange of knowledge between multinational e-commerce business owners	A31	[36]
		Collaborative planning among multinational e-commerce business owners	A32	[60]
		Interactive collaboration among multinational e-commerce partners	A33	[54,61]
	Information sharing among partners.	Create a positive business image for fairness	A34	[51]
		Maintaining agreements with multinational e-commerce partners in information sharing.	A35	[52]
		Integrity practices among partners for real-time information exchange	A36	[53]

### 2.2 Criteria identification

For ranking the identified factors, a total of 5 criteria are identified by thoroughly reviewing most recent studies on supply chain resilience as shown in Table 2.

**Table 2:** Criteria identification.

Index	Criteria	Description
C1	Impact on supply chain resilience	This criterion measures how much of a positive impact the critical factor has on the overall resilience of the supply chain. A factor with a high impact on supply chain resilience will help the supply chain to better withstand and recover from disruptions [62].
C2	Difficulty of implementation	This criterion measures how difficult it is to implement the critical factor. Factors that are more difficult to implement may require more time, resources, and expertise [63].
C3	Cost of implementation	This criterion measures the financial cost associated with implementing the critical factor. Factors that are more expensive to implement may require a significant investment of resources [20].
C4	Time required for implementation	This criterion measures the amount of time required to implement the critical factor. Factors that take longer to implement may require a phased approach or a significant commitment of time and resources [64].
C5	Stakeholder support	This criterion measures the level of support for implementing the critical factor from key stakeholders, such as customers, suppliers, and employees. Factors that have high stakeholder support will be easier to implement and more likely to be successful [56].

## 3 Research methodology

**Fig. 1:**
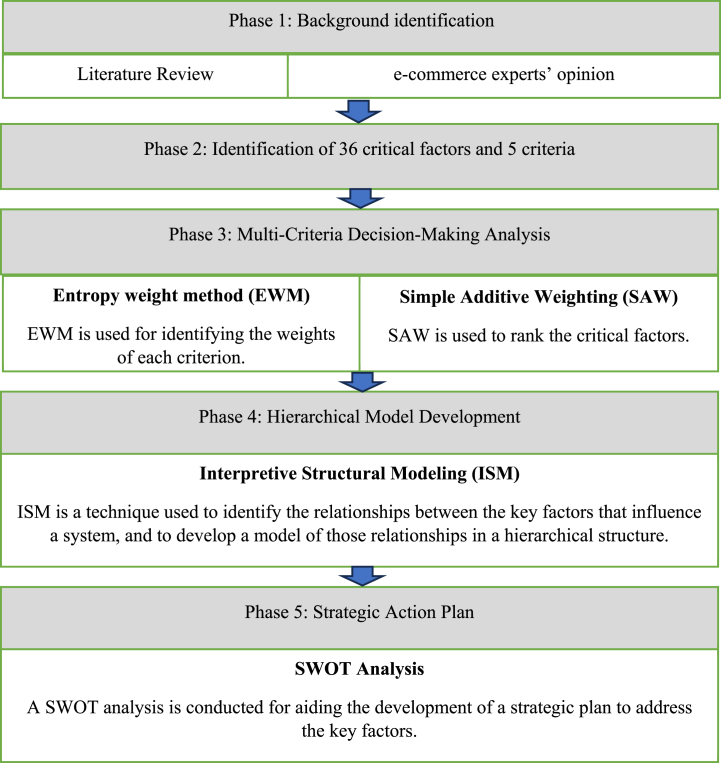
Research methodology.

The research methodology involved four phases (as shown in Fig. 1) to investigate critical factors related to TNSCRE. **Phase 1** comprised a comprehensive literature review, consulting e-commerce experts, and previous studies to identify challenges affecting TEC resilience. In **Phase 2**, 36 critical factors were identified, and five criteria are defined for ranking these factors. **Phase 3** utilized an integrated ENTROPY-SAW technique to quantitatively assess these factors' impact on TNSCRE. In **Phase 4**, the ISM method was employed to understand complex relationships among top-ranked factors. Lastly, in **Phase 5**, a SWOT analysis is conducted to guide TEC business owners in Strategic Action Planning to enhance overall resilience.

### 3.1 Data collection

This study aimed to improve the TEC supply chain's overall resilience by reducing the impact of key critical factors. A comprehensive literature review was conducted to achieve this goal, and experts from the Dhaka, Bangladesh e-commerce industry were consulted. The most important factors that affect the resilience of TEC were chosen for analysis. Additionally, a series of brainstorming sessions involving five industry experts and two academic experts were conducted to refine the ideas gathered from the literature review. Participant details are presented in Table 3.

**Table 3:** Demographic details of participants.

Participant demographics	
Profession/Industry	E-commerce professionals based in Dhaka, Bangladesh
Expertise	•Subject matter experts (Industry oriented) •Academic Scholars (Researchers)
Participants Number	•50 Subject matter experts for the questionnaire •A group of 30 industry and academic experts who specialize in reliability and consistency assessment.
Location	Dhaka, Bangladesh
Expert opinion	•Experts used a five-point rating scale to evaluate the factors. •Experts used linguistic terms such as “very low” to “very high influence” to assess the factors.

The study assessed the validity and reliability of the questionnaire by conducting a reliability analysis using a five-point rating scale to evaluate the importance and feasibility of the identified factors. Further, 50 field experts were asked for feedback on the questionnaire. The questionnaire was created using linguistic terms such as “very low” to “very high influence” and was reviewed by 30 industry professionals, who provided feedback on the identified factors of TNSCRE. The study used Kappa statistics to measure the consistency of the factors, based on the ratings of 30 industry and academic specialists who evaluated the presence of 36 key factors across five areas: management, information, integration, production, and environment. The Kappa index (0.21–0.40) confirmed the reliability of the factors for further research. Further, with validated factors, an ENTROPY-SAW analysis was performed. The Integrated ENTROPY-SAW-ISM approach then revealed interdependencies, indicating the factors' relative importance and impact on fulfilling the study objective.

### 3.2 Data analysis and validation: reliability and consistency test

A survey was conducted with 30 professionals and academicians to determine the feasibility of the critical factors, with a 60 % response rate following Malhotra and Grover [65]. The reliability of the data was assessed using Cronbach's alpha in SPSS-23, resulting in a score of 0.814, which indicates that the data is reliable as shown in the supplementary file Table A1. The mean and standard deviation of the responses were also computed. These statistics showed the importance level of the critical factors based on their mean values. The top 15 influencing factors of TNSCRE in order of ranking were: A14> A33 > A13 > A31 > A12 > A34 > A32 > A11 > A7 > A9 > A23 > A30 > A16 > A19 > A21. These barriers were more significant in achieving resilience in the Transnational supply chain. To ensure that the factors identified were consistent across different perspectives, the study used Kappa statistics, a statistical measure of agreement developed by Cohen [66]. The five perspectives considered were managerial (P1), information (P2), integration (P3), production (P4), and environmental (P5). These perspectives were adopted by Tyagi et al. [67], Sim et al. [68] and Al Amin et al. [69], who added the environmental perspective to enhance sustainability performance. The study used Kappa statistics to measure the consistency of each factor from five perspectives. The results can be found in the supplementary file Table A2, which shows the number of experts who helped identify the factors in each category. Landis and Koch [37] introduced a scale for interpreting Kappa values, indicating that values above 0.21 represent consistency. This study found that the Kappa statistic was ‘k = 0.2290′, which falls within the range (0.21–0.40), signifying a fair degree of consistency among the critical factors in each category. The primary factors were ranked using the ENTROPY-SAW method, and the interrelationships among the sub-factors were analyzed using the ISM approach, as outlined in the methodology section.

## 4 Application of MCDM method

### 4.1 EWM weight calculation

The concept of entropy is well-suited to measure the relative importance of criteria in decision-making [70]. The EWM has been widely applied in supply chain analysis to determine the weights of various criteria, as mentioned in the introduction section. In this research, the EWM is adopted to determine the weights of five benefit-type criteria, calculated by following the below procedure:

Given *m* alternatives to evaluate and *n* evaluation criteria, the initial decision matrix is calculated as follows:

$$D={\left(x_{ij}\right)}_{m\times n}$$

The decision matrix is normalized as:

$$p_{ij}=x_{ij}/\sum_{i=1}^{m}\;x_{ij}$$

The information entropy for each index is defined as:

$$E_{j}=‐{\left(\ln\;m\right)}^{‐1}\sum_{i=1}^{m}\;p_{\text{ij}}\;\ln\;p_{\text{ij}}$$

and the weight obtained from information entropy is calculated as follows:

$$\begin{array}{c} w_{j}=\left(1-E_{j}\right)/\left(n-\sum_{j=1}^{n}\;E_{j}\right)\\ \;\text{where}\;0\leq w_{j}\leq 1\;\text{and}\;\sum_{j=1}^{n}\;w_{j}=1\text{.} \end{array}$$

### 4.2 SAW methodology

The SAW is a multi-criteria decision-making method that is a popular choice for various decision-making problems [12] and used to assess critical factors based on a defined set of criteria. SAW methodology begins by normalizing the decision matrix to be consistent across all criteria. The normalized values are then multiplied by the weights of the criteria, and the weighted values are summed to produce a total score for each alternative. The alternative with the highest total score is ranked as the best alternative. The following basic steps are involved in the SAW method.

1. **Identifying the factors:** The first step is to identify the criteria which is done through the extensive literature review process in the previous section.
2. **Normalize the decision matrix**: The next step is to normalize the values of the decision matrix obtained from the expert opinions. The below formulas are used for this process:

$$Criterion\;is\;a\;benefit\;criterion:r_{ij}^{+}=\frac{X_{ij}}{X_{j}^{\mathrm{Max}}},i=1,2,\ldots ,m$$

$$criterion\;is\;a\;cost\;criterion:r_{ij}^{-}=\frac{X_{j}^{\mathrm{Min}}}{X_{ij}},i=1,2,\ldots ,m$$

3. **Assigning weights to the factors:** The weights of the factors reflect their importance in the decision-making process. The weights are assigned by the EWM.
4. **Calculating weighted normalized matrix:** The weighted criteria values are calculated by multiplying the normalized criteria values by the weights of the criteria. According to the following relationship, the normalized matrix is multiplied by the weight of the criteria:

$$v_{ij}\left(x\right)=w_{j}r_{ij}\left(x\right)\;i=1,\ldots ,m\;;j=1,\ldots ,n$$

5. **Calculating the total score for each alternative:** The total score for each factor is calculated by summing the weighted criteria values using the below formula:

$$S_{i}=\sum_{j=1}^{n}v_{ij}$$

6. **Ranking the alternatives**: The factors are ranked based on their total scores, with the factor with the highest total score ranked as the top critical factor and the remaining factors are ranked in descending order.

### 4.3 Interpretive structural model

This research uses the ISM approach to assess the factors that influence the resilience of TNSCRE. ISM is a technique that is used to break down complex problems into smaller components by obtaining the expertise of specialists. The ISM method was utilized for this study because it offers several advantages, including (i) It can identify and map the interrelationships between different factors. (ii) It can reveal the overall structure of a system based on a set of factors [71]. (iii) It can be used to create a directed graph model that visualizes the relationships between the different elements of a system [72]. (iv) It can help to determine the direction and sequence of complex interactions between different factors in a system [73].

The following lists the numerous steps involved in the ISM approach.

Step 1 Factors Influencing Transnational e-commerce supply chain resilience are gathered.
Step 2 Using the factors gathered in step 1, a logical relationship between the factors is identified.
Step 3 Structural Self-Interaction Matrix (SSIM) is formed for factors; this confirms pairwise links between framework factor components.
Step 4 Transitivity check of the SSIM and form Final reachability matrix (FRM)
Step 5 Develop Level partition from the reachability matrix which is achieved in step 4.
Step 6 An ISM factors digraph is drawn, which shows the ISM structure and the interconnections of factors at different levels.

## 5 Numerical illustrations

### 5.1 Calculation of the EW of criteria

The EWM is a widely used objective method for determining the weights of attributes in objective functions used in decision-making algorithms. The EWM is an objective method, meaning that it does not require any subjective input from the decision-maker. The EWM works by first calculating the entropy of each criterion. Entropy is a measure of the uncertainty in a system. The higher the entropy of a criterion, the more uncertain it is. The EWM then assigns a weight to each criterion based on its entropy. Criteria with higher entropy are given higher weights. Following the entropy steps outlined in Section 4.1, the calculated weights of the five criteria are shown in Table 4.

**Table 4:** Weight of criteria.

Criteria	C1	C2	C3	C4	C5
Weight	0.18068	0.24949	0.20101	0.19233	0.1765

### 5.2 Ranking the factors through SAW analysis

In the context of ranking critical factors of TNSCRE, the SAW method is used to rank factors based on their importance and performance. To do this, the first step is to identify the relevant criteria for ranking the factors. Once the criteria have been identified, weights are assigned to each criterion based on its importance using EWM. The next step is to normalize the data for each factor so that all factors are on the same scale. The normalized data is then multiplied by the corresponding weights to calculate the weighted normalized scores. Finally, the weighted normalized scores for each factor are summed to calculate the total weighted normalized scores. The factors are then ranked based on their total weighted normalized scores. Table 5 shows detailed calculations of the decision matrix, normalized matrix and weighted normalized matrix based on steps 1–6 mentioned in the methodology section.

**Table 5:** Decision matrix, normalized matrix, weighted normalized matrix and rank of factors.

	Decision Matrix					Normalized Matrix					Weighted Normalized Matrix					Ranking	
	C1	C2	C3	C4	C5	C1	C2	C3	C4	C5	C1	C2	C3	C4	C5	Si	Rank
A1	2	1	1	1	1	0.4	0.2	0.2	0.2	0.2	0.072	0.05	0.04	0.038	0.035	0.236	17
A2	2	1	1	1	1	0.4	0.2	0.2	0.2	0.2	0.072	0.05	0.04	0.038	0.035	0.236	17
A3	1	1	1	2	1	0.2	0.2	0.2	0.4	0.2	0.036	0.05	0.04	0.077	0.035	0.238	16
A4	1	1	1	1	1	0.2	0.2	0.2	0.2	0.2	0.036	0.05	0.04	0.038	0.035	0.2	18
A5	1	1	1	1	1	0.2	0.2	0.2	0.2	0.2	0.036	0.05	0.04	0.038	0.035	0.2	18
A6	1	1	1	1	1	0.2	0.2	0.2	0.2	0.2	0.036	0.05	0.04	0.038	0.035	0.2	18
A7	4	3	3	2	2	0.8	0.6	0.6	0.4	0.4	0.145	0.15	0.121	0.077	0.071	0.562	9
A8	1	1	1	1	1	0.2	0.2	0.2	0.2	0.2	0.036	0.05	0.04	0.038	0.035	0.2	18
A9	3	3	3	2	2	0.6	0.6	0.6	0.4	0.4	0.108	0.15	0.121	0.077	0.071	0.526	10
A10	1	1	1	1	1	0.2	0.2	0.2	0.2	0.2	0.036	0.05	0.04	0.038	0.035	0.2	18
A11	4	3	3	3	3	0.8	0.6	0.6	0.6	0.6	0.145	0.15	0.121	0.115	0.106	0.636	8
A12	5	4	4	3	3	1	0.8	0.8	0.6	0.6	0.181	0.2	0.161	0.115	0.106	0.762	5
A13	5	5	4	4	4	1	1	0.8	0.8	0.8	0.181	0.249	0.161	0.154	0.141	0.886	3
A14	5	5	5	5	5	1	1	1	1	1	0.181	0.249	0.201	0.192	0.176	1	1
A15	1	1	1	1	1	0.2	0.2	0.2	0.2	0.2	0.036	0.05	0.04	0.038	0.035	0.2	18
A16	3	2	2	1	1	0.6	0.4	0.4	0.2	0.2	0.108	0.1	0.08	0.038	0.035	0.362	13
A17	1	1	1	1	1	0.2	0.2	0.2	0.2	0.2	0.036	0.05	0.04	0.038	0.035	0.2	18
A18	1	1	1	1	1	0.2	0.2	0.2	0.2	0.2	0.036	0.05	0.04	0.038	0.035	0.2	18
A19	2	2	2	1	1	0.4	0.4	0.4	0.2	0.2	0.072	0.1	0.08	0.038	0.035	0.326	14
A20	1	1	1	1	1	0.2	0.2	0.2	0.2	0.2	0.036	0.05	0.04	0.038	0.035	0.2	18
A21	2	2	1	1	1	0.4	0.4	0.2	0.2	0.2	0.072	0.1	0.04	0.038	0.035	0.286	15
A22	1	1	1	1	1	0.2	0.2	0.2	0.2	0.2	0.036	0.05	0.04	0.038	0.035	0.2	18
A23	3	3	2	2	2	0.6	0.6	0.4	0.4	0.4	0.108	0.15	0.08	0.077	0.071	0.486	11
A24	1	1	1	1	1	0.2	0.2	0.2	0.2	0.2	0.036	0.05	0.04	0.038	0.035	0.2	18
A25	1	1	1	1	1	0.2	0.2	0.2	0.2	0.2	0.036	0.05	0.04	0.038	0.035	0.2	18
A26	1	1	1	1	1	0.2	0.2	0.2	0.2	0.2	0.036	0.05	0.04	0.038	0.035	0.2	18
A27	1	1	1	1	1	0.2	0.2	0.2	0.2	0.2	0.036	0.05	0.04	0.038	0.035	0.2	18
A28	1	1	1	1	1	0.2	0.2	0.2	0.2	0.2	0.036	0.05	0.04	0.038	0.035	0.2	18
A29	1	1	1	1	1	0.2	0.2	0.2	0.2	0.2	0.036	0.05	0.04	0.038	0.035	0.2	18
A30	3	2	2	2	2	0.6	0.4	0.4	0.4	0.4	0.108	0.1	0.08	0.077	0.071	0.436	12
A31	5	4	4	4	4	1	0.8	0.8	0.8	0.8	0.181	0.2	0.161	0.154	0.141	0.836	4
A32	4	4	3	3	3	0.8	0.8	0.6	0.6	0.6	0.145	0.2	0.121	0.115	0.106	0.686	7
A33	5	5	5	4	4	1	1	1	0.8	0.8	0.181	0.249	0.201	0.154	0.141	0.926	2
A34	4	4	4	3	3	0.8	0.8	0.8	0.6	0.6	0.145	0.2	0.161	0.115	0.106	0.726	6
A35	1	1	1	1	1	0.2	0.2	0.2	0.2	0.2	0.036	0.05	0.04	0.038	0.035	0.2	18
A36	1	1	1	1	1	0.2	0.2	0.2	0.2	0.2	0.036	0.05	0.04	0.038	0.035	0.2	18

**Fig. 2:**
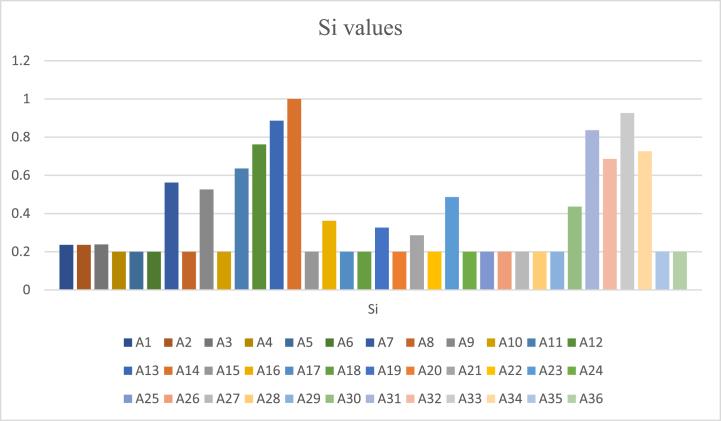
Si values of each critical factor.

The following Fig. 2 shows the amounts of $S_{i}$ of each factor. The factor with the highest Si score is ranked as the top critical factor, and the remaining factors are ranked in descending order. The total score for each factor is calculated by summing the weighted criteria values. This means some criteria are more important than others, depending on the situation. By understanding the Si scores of different factors, stakeholders will better identify the most important factors to focus on to improve TEC's overall resilience.

### 5.3 ISM analysis

This study selects the top 15 factors for ISM analysis after ranking them using SAW based on their relative importance identified through literature reviews, reliability test and interdependence in the overall system. To focus on critical factors and comprehensibility, taking top-ranked factors for ISM model is important. Another reason is to identify more concise hierarchical relationships among these selected factors. With the help of 15 academics and e-commerce experts, contextual linkages between the elements are constructed. This study used a focused group discussion technique to gather data. ISM recommends using a sample size of 10–30 survey participants to achieve reliable results [55,74]. Our study involved five academics and ten e-commerce businesspeople in performing the ISM analysis.

#### 5.3.1 Structural Self-Interaction Matrix (SSIM)

The SSIM is calculated by comparing each pair of sub-factors listed in Table 6. The qualitative concept of “distance” is used to determine the relationship between the factors, which means, one factor causes another factor to impact the SCRE. If this is not the case, then the factors are not compatible. Each factor's connectedness is assessed by examining whether there is a relationship between it and any other factor (i and j), and if so, how that relationship functions. The relationship between factors (i and j) is indicated by a four-letter code (V: Factor i influences Factors j; A: Factor j influences Factors i; X: Factor i and j influence each other; and O: Factor i and j are unrelated).

**Table 6:** SSIM for top 15 influencing factors.

Variables	1	2	3	4	5	6	7	8	9	10	11	12	13	14	15
1		O	X	O	O	O	O	O	A	A	O	O	O	V	V
2			A	O	O	V	O	A	O	O	O	V	O	O	O
3				O	O	O	O	O	V	V	O	V	O	V	V
4					O	X	V	O	O	O	O	O	O	O	O
5						O	O	V	V	V	O	O	O	O	O
6							V	A	O	O	O	O	O	O	O
7								O	O	O	A	O	O	O	O
8									V	V	V	O	O	O	O
9										O	O	O	O	O	O
10											O	O	O	O	O
11												O	O	O	O
12													O	O	O
13														O	O
14															O
15															

#### 5.3.2 Final reachability matrix

To convert SSIM into reachability matrix, each cell's component has been changed to binary digits (0 and 1), as displayed in Table 7. This transformation is done based on this system.

a) If the component of cell (i, j) in the SSIM is V, then (i, j) component of the cell = 1 and the (j,i) component = 0 in the reachability matrix.
b) If the component of the cell (i, j) is A, then (i,j) component becomes 0 and (j, i) component becomes 1.
c) If the component of the cell (i, j) X, then both the cells (i, j) and (j,i) become 1.
d) If the component of the cell (i, j) O, then the entries in both the cells (i, j) and (j,i) become 0.

**Table 7:** Reachability matrix.

Variables	1	2	3	4	5	6	7	8	9	10	11	12	13	14	15	Driving Power
1	1	1*	1	1*	0	1*	1*	0	1*	1*	0	1*	0	1	1	11
2	0	1	0	1*	0	1	1*	0	0	0	0	1	0	0	0	5
3	1	1	1	1*	0	1*	1*	0	1	1	0	1	0	1	1	11
4	0	0	0	1	0	1	1	0	0	0	0	0	0	0	0	3
5	1*	1*	1*	1*	1	1*	1*	1	1	1	1*	1*	0	1*	1*	14
6	0	0	0	1	0	1	1	0	0	0	0	0	0	0	0	3
7	0	0	0	0	0	0	1	0	0	0	0	0	0	0	0	1
8	1*	1	1*	1*	0	1	1*	1	1	1	1	1*	0	1*	1*	13
9	1	1*	1*	1*	0	1*	1*	0	1	1*	0	1*	0	1*	1*	11
10	1	1*	1*	1*	0	1*	1*	0	1*	1	0	1*	0	1*	1*	11
11	0	0	0	0	0	0	1	0	0	0	1	0	0	0	0	2
12	0	0	0	0	0	0	0	0	0	0	0	1	0	0	0	1
13	0	0	0	0	0	0	0	0	0	0	0	0	1	0	0	1
14	0	0	0	0	0	0	0	0	0	0	0	0	0	1	0	1
15	0	0	0	0	0	0	0	0	0	0	0	0	0	0	1	1
Dependence Power	6	7	6	9	1	9	11	2	6	6	3	8	1	7	7	

#### 5.3.3 Level partitions

We need to assign levels to each of the identified factors. Once we have created the reachability matrix, we can determine each factor's reachability and antecedent set. The antecedent set contains the factors that support the integration of the factor being considered and the factors that can be used to expand upon. The intersection set of the antecedent set and the reachability set contains all of the factors that are both reachable from and antecedent to the factor being considered. These factors are placed at the highest level of the ISM model. The process is repeated until all of the factors have been assigned a level. The level partitioning iterations are shown in the supplementary file (Table B1-B7) attached to the manuscript to reduce the length of the article. The final level partitions of the factors are presented in Table 8.

**Table 8:** Final level partitioning of factors.

Elements (Mi)	Reachability Set R (Mi)	Antecedent Set A (Ni)	Intersection Set R (Mi)∩A (Ni)	Level
1	1, 3, 9, 10	1, 3, 5, 8, 9, 10	1, 3, 9, 10	4
2	2	1, 2, 3, 5, 8, 9, 10	2	3
3	1, 3, 9, 10	1, 3, 5, 8, 9, 10	1, 3, 9, 10	4
4	4, 6	1, 2, 3, 4, 5, 6, 8, 9, 10	4, 6	2
5	5	5	5	6
6	4, 6	1, 2, 3, 4, 5, 6, 8, 9, 10	4, 6	2
7	7	1, 2, 3, 4, 5, 6, 7, 8, 9, 10, 11	7	1
8	8	5, 8	8	5
9	1, 3, 9, 10	1, 3, 5, 8, 9, 10	1, 3, 9, 10	4
10	1, 3, 9, 10	1, 3, 5, 8, 9, 10	1, 3, 9, 10	4
11	11	5, 8, 11	11	2
12	12	1, 2, 3, 5, 8, 9, 10, 12	12	1
13	13	13	13	1
14	14	1, 3, 5, 8, 9, 10, 14	14	1
15	15	1, 3, 5, 8, 9, 10, 15	15	1

#### 5.3.4 Reduced conical matrix

The level partitioning is sorted and simplified to form the Reduced conical matrix. A reduced conical matrix helps in forming the ISM digraph as shown in Table 9.

**Table 9:** Reduced conical matrix.

Variables	7	12	13	14	15	4	6	11	2	1	3	9	10	8	5	Driving Power	Level
7	1	0	0	0	0	0	0	0	0	0	0	0	0	0	0	1	1
12	0	1	0	0	0	0	0	0	0	0	0	0	0	0	0	1	1
13	0	0	1	0	0	0	0	0	0	0	0	0	0	0	0	1	1
14	0	0	0	1	0	0	0	0	0	0	0	0	0	0	0	1	1
15	0	0	0	0	1	0	0	0	0	0	0	0	0	0	0	1	1
4	1	0	0	0	0	1	1	0	0	0	0	0	0	0	0	3	2
6	1	0	0	0	0	1	1	0	0	0	0	0	0	0	0	3	2
11	1	0	0	0	0	0	0	1	0	0	0	0	0	0	0	2	2
2	0	1	0	0	0	1*	1	0	1	0	0	0	0	0	0	5	3
1	0	0	0	1	1	0	0	0	1*	1	1	1*	1*	0	0	11	4
3	0	0	0	1	1	0	0	0	1	1	1	1	1	0	0	11	4
9	0	0	0	1*	1*	0	0	0	1*	1	1*	1	1*	0	0	11	4
10	0	0	0	1*	1*	0	0	0	1*	1	1*	1*	1	0	0	11	4
8	0	0	0	0	0	0	0	1	0	1*	1*	1	1	1	0	13	5
5	0	0	0	0	0	0	0	0	0	0	0	0	0	1	1	14	6
Dependence Power	11	8	1	7	7	9	9	3	7	6	6	6	6	2	1		
Level	1	1	1	1	1	2	2	2	3	4	4	4	4	5	6		

## 6 Result and discussion

This study employed a two-step approach to identify and analyze the key factors influencing the TNSCRE. First, based on the fundamental idea of SCRE, we identified 36 potential influencing factors. These factors were then categorized into three primary groups by the framework: evolution capability, efficiency, and supply chain adaptability. We utilized the SAW technique to prioritize the most impactful factors for further analysis. This technique allowed us to rank all 36 factors based on their relative importance, as established through a comprehensive literature review. Following the SAW analysis, the top 15 most significant factors were selected for further investigation using the ISM approach (detailed in Table 6). Two key reasons motivated this selection. Firstly, the SAW analysis highlighted these factors' relative importance within the overall TNSCRE framework. Secondly, focusing on a smaller set of 15 factors enabled us to establish a more concise and interpretable hierarchical structure of their interdependencies within the system. Due to the emphasis on interpretability and manageability in ISM analysis, many research articles focus on 10 to 15 factors [75,76]**.** This facilitated a deeper understanding of the driving and dependent factors influencing TNSCRE. Finally, based on the insights gained from the ISM analysis, a SWOT analysis was conducted to identify potential strategic measures for enhancing the recovery speed of supply chain interruptions, a key factor identified through the analysis.

### 6.1 ISM based conceptual model

**Fig. 3:**
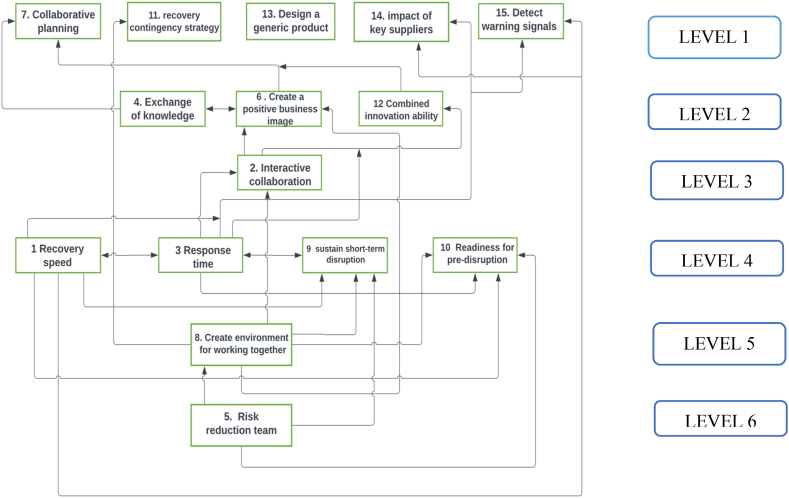
ISM digraph of influencing factors.

The ISM model of influencing factors is created by analyzing the reachability matrix, which shows the relationships between different factors. Transitivity, which is the indirect influence of one factor on another through a third factor, is removed to create the ISM model as depicted in Fig. 3. Upward arrows in the ISM model indicate that one factor directly influences another.

### 6.2 MICMAC analysis

**Fig. 4:**
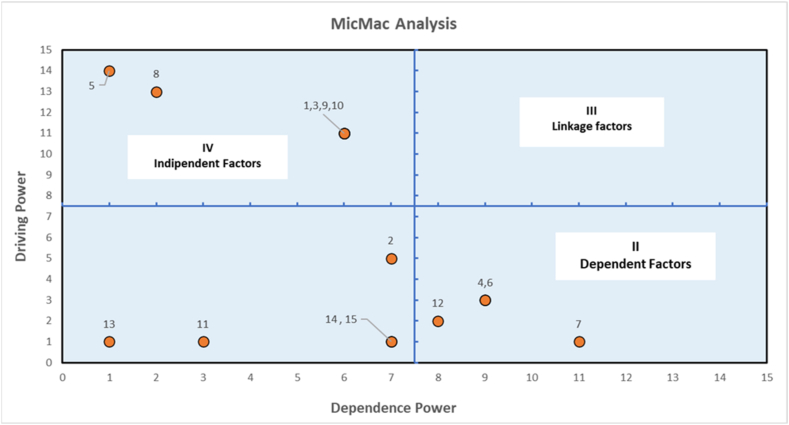
Micmac Analysis based on driving and dependence power.

The fundamental goal of a MICMAC is to assess the influence and interdependence of multiple factors. The factors are segmented into four groups according to their driving and dependent powers. They are Independent, Dependent, Linkage, and Autonomous. The diagram shows the factors driving and dependent powers in Fig. 4. The factors in quadrants-I are autonomous. Dependence and independent factors are displayed in quadrants II and IV. There are no factors in the linkage section.

### 6.3 Sensitivity analysis

**Fig. 5:**
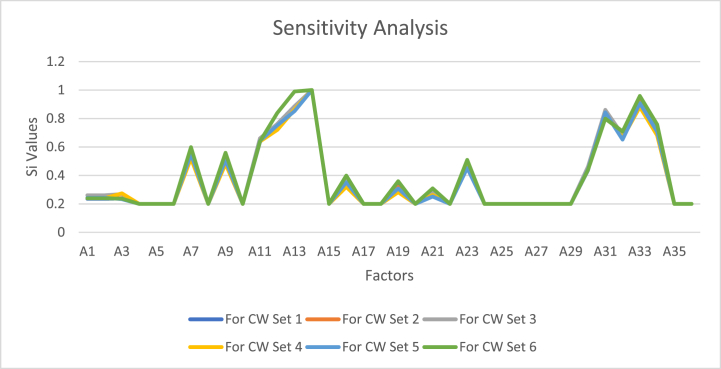
Sensitivity analysis.

Table 10 presents the 6 sets of criteria weight based on the EWM method and Fig. 5 illustrates the sensitivity of various factors denoted as A1 to A36 concerning changes in criteria weight (CW) across six distinct CW sets (See Table C1 in supplementary file).

**Table 10:** Set of criteria weight based on EWM method.

Criteria (C)					
Weight (W)	C1	C2	C3	C4	C5
Set 1	0.18068	0.24949	0.20101	0.19233	0.1765
Set 2	0.20	0.20	0.20	0.20	0.20
Set 3	0.30665	0.11603	0.079125	0.34597	0.15222
Set 4	0.21573	0.043276	0.28293	0.16901	0.28905
Set 5	0.25686	0.29901	0.27521	0.072722	0.09619
Set 6	0.19688	0.34914	0.25018	0.18567	0.018123

These Si values, which represent the ranking of factors, provide a means to assess their relative importance, with higher Si values signifying a higher rank. This analysis reveals that the Si values for individual factors fluctuate in response to changes in criteria weight. For instance, the Si value for factor A13 varies from 0.88 for CW Set 1 to 0.99 for CW Set 6, indicating its sensitivity to changes in criteria weight. Importantly, this figure also demonstrates that ranking factors can shift in response to such changes. For instance, factor A12 is ranked as the 5th most important factor in CW Set 1 but becomes the 6th most important in CW Set 6, highlighting the dynamic nature of these rankings based on the criteria weight set used.

One notable pattern that emerges from this analysis is that the factors most sensitive to changes in criteria weight are those exhibiting significant differences in importance across different CW sets. Factor A13, for instance, demonstrates the highest sensitivity between CW Set 1 and CW Set 6, with a difference of 0.11 in its Si value as criteria weight varies. The criteria weight represents the relative significance of a criterion in the decision-making process, where a higher criteria weight implies greater importance. As observed, criteria weight changes substantially impact the ranking of factors, further emphasizing the dynamic nature of decision-making models. This sensitivity analysis offers valuable insights into the factors most sensitive to criteria weight changes, aiding in resource allocation, robust decision-making model development, and identifying critical factors for achieving specific goals. For instance, factor A14 consistently ranks first in every sensitivity analysis, underscoring its importance in supply chain decision-making. In conclusion, sensitivity analysis is a valuable tool to enhance informed decision-making across various applications.

### 6.4 Strategic action plan using SWOT analysis

**Fig. 6:**
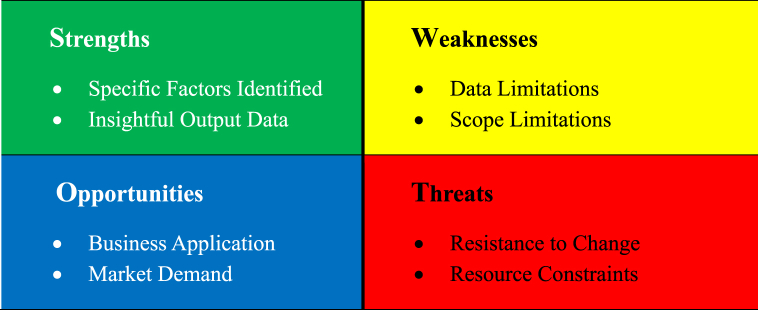
SWOT analysis for the strategic action plan.

A SWOT analysis is conducted to provide a strategic action plan based on the findings of the research on transnational e-commerce supply chain resilience as shown in Fig. 6. It allows organizations to identify their internal and external environment and to develop strategies that leverage their strengths and opportunities while mitigating their weaknesses and threats [77].

SWOT analysis applicability depends on each organization's unique context. Our 15 key factors can't be universally categorized as strengths, weaknesses, opportunities, or threats. Organizations can leverage our findings within a SWOT framework to craft specific strategic plans. Businesses can enhance their supply chain resilience in the context of cross-border e-commerce by doing a SWOT analysis in conjunction with our research findings. Our research demonstrates a clear set of strengths, including providing insightful data on the critical factors influencing supply chain resilience, focusing on key aspects such as disruption recovery speed, interactive collaboration, and response time to supply chain disruptions. However, it is critical to recognize the weaknesses such as geographical limitations on data and the scope of our research, which did not cover all aspects of cross-border e-commerce supply chain resilience.

In terms of opportunities, firms can use the research findings to enhance their operations and fulfill the expanding market need for resilient supply chains, giving them a competitive advantage. Nonetheless, applying these insights could involve certain risks, such as organizational resistance to change or limitation of resources hindering effective implementation. Businesses can navigate these challenges and seize opportunities by implementing a strategic action plan that includes capitalizing on strengths, addressing weaknesses through additional data acquisition or collaboration, taking advantage of identified opportunities, and mitigating potential threats like resistance to change. Our study has shown that interactive engagement with partners and suppliers is critical. Finally, organizations should prioritize, implement, and regularly review their action plans, ensuring that they are aligned with their broader objectives for long-term development in the globalized landscape.

### 6.5 Findings, interpretation and insights

This study's findings may shed light on the following areas for transnational e-commerce businesses.

(a) TEC businesses may think about enhancing SCRE in view of the major influencing factors.

The major influencing elements significantly influence the system due to their great dominance to affect other elements. The top-ranked and most important factors affecting the TNSCRE are the recovery speed of the supply chain, interactive collaboration among multinational e-commerce partners, response time of supply chain disruption, exchange of knowledge between multinational e-commerce partners, Collaborative risk reduction team, create a positive business image for fairness, Collaborative planning among TEC partner, and create an environment where partners can work together. As a result, the following are the top 15 influencing factors of TNSCRE in order of ranking done by SAW: **A14** > **A33 > A13 > A31 > A12 > A34 > A32 > A11 > A7** > **A9** > **A23 > A30 > A16 > A19 > A21.**

(b) How do the key critical factors interact with one another? In what ways does the SCRE suffer?

The interaction analysis of the key critical factors in SCRE can reveal the answers to these queries. The analysis of key critical factors in SCRE involves clustering them into four groups: independent, dependent, linking, and autonomous factors. Independent factors, characterized by their significant driving power and minimal dependency, directly influence other system variables, such as the potential to sustain short-term disruptions and collaborative risk reduction teams. On the other hand, dependent factors are influenced by other factors but heavily influence them in return, as seen in examples like combined innovation ability and collaborative planning among multinational e-commerce partners. Linking factors, while not identified in this study, typically strongly influence other factors in the system and are influenced by them, creating ripple effects across the system. With weak driving and dependency power, autonomous factors operate relatively independently from the system, encompassing aspects like collaborative planning and assessing key suppliers' impact. This framework provides a nuanced understanding of the intricate interactions within the SCRE domain, facilitating targeted interventions to enhance supply chain resilience.

(c) TEC companies can improve SCRE by optimizing their supply networks based on the hierarchical relationships between different factors. This can be done by following these suggestions:
  • In order to increase the TNSCRE, the supply chain's adaptability is the most crucial and significant component. Businesses can further promote growth by focusing on the risk-managing team, utilizing risk-handling experience, and creating an environment where partners can work together.
  • Supply chain evolution which directly influences the growth of TNSCRE. Three factors can be taken into account: the ability of international e-commerce partners to coordinate, the development of an environment of cooperation among partners, and create a positive business image for fairness.
  • One of the major factors that directly affect the development of TNSCRE is the supply chain's efficiency. By focusing on the rate of recovery from supply chain interruptions, the time it takes to respond to interruptions, and coordinated planning among international e-commerce partners, we can make the establishment of supply chains more agile.
  • The most efficient strategies to enhance the TNSCRE are to increase the supply chain's adaptability and capacity for improvement of efficiency and evolution. The emergency plan, contingency planning, knowledge management, and trust among stakeholders cause the most significant impact on the TNSCRE. Specific factors that significantly affect the TNSCRE include the use of multiple international suppliers, redundant transnational logistics and storage systems, safety inventory, and an efficient risk management department.
(d) After conducting a thorough analysis using the SAW– ISM method, it was found that the recovery speed of the supply chain ranked as the top priority for improvement. In order to create an effective strategy for reducing the negative impact on supply chain resilience, a SWOT analysis was constructed to visually represent the root causes of the problem and to provide a clear plan of action for addressing them.

## 7 Conclusion

This study developed a hybrid model to investigate the factors affecting TNSCRE by integrating the EWM, SAW, and ISM. The study identified 36 critical factors categorized under three dimensions: supply chain adaptability, supply chain efficiency, and supply chain evolution. These factors were then ranked using the EWM and SAW methods. Sensitivity analysis revealed that the findings were robust to changes in the weights of the criteria. The ISM analysis mapped the interrelationships among the key factors, highlighting the significance of factors such as the speed of supply chain disruption recovery, interactive collaboration, and response time to supply chain disruption. A SWOT analysis was also conducted to develop a strategic action plan for addressing these significant factors. The findings suggest that companies can improve their TNSCRE by focusing on the following areas:

• Building a more adaptable supply chain: This includes developing the ability to respond to demand, supply, and regulations changes quickly and efficiently.
• Improving supply chain efficiency involves streamlining operations, reducing costs, and improving visibility and traceability throughout the supply chain.
• Investing in supply chain evolution includes embracing new technologies, developing new business models, and forging new partnerships.

The findings of this study can help companies improve their existing supply chain resilience and achieve sustainable growth in the globalization context, particularly in the current environment, where transnational e-commerce supply chains face various challenges, including the COVID-19 pandemic, the ongoing conflict in Ukraine, and rising inflation. By focusing on the factors identified in this study, companies can better prepare for and respond to these challenges, ensuring the resilience of their supply chains and the continued success of their businesses.

### 7.1 Contributions of this research

This article makes a significant contribution in several ways. Initially, it innovatively incorporates the core SCRE theory, unlike earlier studies that often focused on one aspect of supply network efficiency (capability of adaptation or capability of evolution) rather than considering all three together. The research aims to thoroughly investigate the interplay between these three components, paving the way for new avenues of inquiry. Furthermore, the study creates an integrated system that encompasses 36 influencing elements of TNSCRE across different tiers based on the foundational SCRE theory. It places primary emphasis on discussing how these influencing factors interact, using the SAW method to rank them. Previous research has typically concentrated on identifying correlations between key determinants and SCRE, neglecting the examination of how these influencing factors interact. This research reveals the method for uncovering these interactions, providing a theoretical framework for future research on rational hierarchies. Subsequently, the research constructs a hierarchy of influencing factors for TNSCRE using the ISM technique, helping elucidate the interactions among contextual factors. Lastly, for the first time in the field of TNSCRE, the study conducts a SWOT analysis to develop a strategic action plan. This plan is designed to assist business owners on how to address the critical factors identified in the study.

### 7.2 Implication of this study

The implications of this research are substantial on both theoretical and practical levels in the field of TNSCRE. The research advances the field by holistically incorporating the core SCRE theory, addressing all aspects of supply network efficiency, thereby offering a more comprehensive understanding of TNSCRE dynamics. Developing an integrated system of 36 influencing factors and using the SAW method to rank them provides a valuable resource for decision-makers to prioritize critical factors affecting TNSCRE. Furthermore, employing the ISM technique to investigate relationships between these aspects adds depth to the field's understanding, assisting in the creation of more effective tactics. The strategic action plan generated through SWOT analysis offers practical guidance for businesses seeking to enhance their supply chain resilience in the global context. Finally, the study emphasizes future research areas, promoting deeper investigation of sophisticated methodologies and the interconnectedness hierarchy of influencing elements. In conclusion, this research significantly contributes to TNSCRE theory and practice, offering valuable insights and tools for scholars and practitioners while paving the way for future research endeavors in this vital field.

### 7.3 Limitations and future directions

This study provides a comprehensive understanding of the factors that impact TNSCRE from the perspective of multiple stakeholders. However, it is important to note that studying has some limitations. First, the SAW approach used to rank the critical factors is somewhat subjective. In future research, the data's subjectivity may only be somewhat reduced by the fuzzification; it cannot be eliminated. Second, the ISM analysis used to evaluate the interrelationships among the key factors does not allow for the quantification of these relationships. Future research can address these limitations by (i) using more sophisticated MCDM methods to assess the influencing elements of TNSCRE, (ii) Exploring and understanding the TNSCRE's inter-connection hierarchy of critical factors using Structural Equation Modeling (SEM), (iii) Developing and testing more comprehensive strategic action plans for addressing the key factors impacting TNSCRE using Fishbone or Pareto analysis. This study provides a valuable foundation for future research on TNSCRE. By addressing the limitations identified above, future research can develop a more comprehensive understanding of the factors that impact TNSCRE and develop more effective strategies for improving TNSCRE.

## Funding

This research received no external funding

## Data availability statement

Data is included in the article/supplementary file. The additional data will be available on reasonable request from the corresponding author.

## Institutional review board statement

The Institutional Review Board of HBKU granted approval for the study under protocol code HBKU-IRB-2024-51 on August 13, 2023.

## CRediT authorship contribution statement

**Dewan Hafiz Nabil:** Writing – original draft, Methodology, Investigation, Formal analysis, Conceptualization. **Md Al Amin:** Writing – review & editing, Writing – original draft, Validation, Methodology, Conceptualization. **Roberto Baldacci:** Writing – review & editing, Supervision.

## Declaration of competing interest

The authors declare that they have no known competing financial interests or personal relationships that could have appeared to influence the work reported in this paper.
