# Supplementary material for: Enhancing resilience in transnational E-commerce supply chains: Critical factors, perspectives and strategic action plan
Source: Heliyon. 2024 May 16;10(10):e31274. doi: 10.1016/j.heliyon.2024.e31274 (PMC11133826; doi:10.1016/j.heliyon.2024.e31274)
Supplement: Multimedia component 1 [file mmc1.docx]

**Supplementary File for E-commerce Resilience**

**Questionnaire guidelines:**

- The survey consists of 15 questions and should take less than 10 minutes to complete.
  If you need any further information, you can directly contact with Md Al Amin at
  [mdal44149@hbku.edu.qa](mailto:mdal44149@hbku.edu.qa).
- The questionnaire entitled “Questionnaire.docx” is attached herewith for the English version. The participants’ IDs will be generated automatically from the system.
- Your responses will be kept completely confidential. You may need to provide an e-mail
  address while you complete the Internet survey. Note your email address will not be stored
  with data from your survey.

**Section 1: Demographics**:

**Q1. Please specify your age group:**

a) Under 18

b) 18-24

c) 25-34

d) 35-44,

e) 45-54

f) 55-65

**Q2. What is your gender?**

a) Male

b) Female

c) Other

d) Prefer not to say

**Q3. Which of the following best describes your professional background or occupation?**

a) E-commerce business owner

b) Supply chain manager

c) Academic researcher

d) Other, please specify

**Section 2: Knowledge and Perception of Transnational E-commerce Supply Chain Resilience (TNSCRE)**

**Q5. Have you heard of the term "Transnational E-commerce Supply Chain Resilience" (TNSCRE) before participating in this survey?**

a) Yes

b) No

**Q6. On a scale of 1 to 5, how familiar are you with the concept of TNSCRE?**

| **Linguistic Variable** | **Very Familiar** | **Familiar** | **Moderate Familiar** | **Less Familiar** | **Not all Familiar** |
| --- | --- | --- | --- | --- | --- |
| Influence Score | 5 | 4 | 3 | 2 | 1 |

**Q7. From your perspective, how critical is TNSCRE in the context of transnational e-commerce?**

a) Not critical at all

b) Somewhat critical

c) Moderately critical

d) Critical

e) Very critical

**Q8. What do you believe are the most crucial factors influencing TNSCRE? (Open-ended).**

**Section 3: Factors Affecting TNSCRE**

**Q9. How important do you consider the speed of supply chain disruption recovery in TNSCRE?**

a) Not important

b) Somewhat important

c) moderately Important

d) Important

e) Very important

**Q10. To what extent do you think interactive collaboration contributes to TNSCRE?**

a) Not at all

b) Slightly

c) Moderately

d) Significantly

e) Very Significantly

**Q11. How critical is the response time to supply chain disruption for TNSCRE?**

a) Not critical at all

b) Somewhat critical

c) Moderately Critical

c) Critical

d) Very critical

**Q12. Please rank the following categories in order of importance in TNSCRE:**

(Supply chain adaptability, Supply chain efficiency, Supply chain evolution) by dragging and dropping them in your preferred order [See Table 1].

**Table 1.** Influencing factors of TNSCRE.

| **Primary** | **Secondary** | **Third Level** | **Index** | **Reference** |
| --- | --- | --- | --- | --- |
| Supply Chain Adaptability | Supply chain flexibility | Acquainted with international electronic-commerce SC management | A1 | [1,39] |
|  |  | Resource flexibility of multinational e-commerce companies | A2 | [64] |
|  |  | SC structure flexibility for multinational e-commerce | A3 | [24,25] |
|  | Supply chain redundancy | Safety stock inventory for multinational e-commerce | A4 | [49] |
|  |  | Multiple vendor procurement | A5 | [50] |
|  |  | Availability of multiple modes of Transnational transport and warehouse capacity | A6 | [50] |
|  | Adaptive management | Capability to cope with long-term disruption. | A7 | [16] |
|  |  | Readiness to handle pre-disruption | A8 | [2] |
|  |  | Potential to sustain short-term disruption | A9 | [28] |
|  | Risk reduction practice | Create an environment where partners can work together | A10 | [52] |
|  |  | Collaborative risk reduction team. | A11 | [52] |
|  |  | Mutual contingency plan among partners | A12 | [53] |
| Supply Chain efficiency | Supply chain agility | Speed of Recovery from disruption | A13 | [29] |
|  |  | Awareness about the supply chain's major links | A14 | [47] |
|  |  | Time required to response in supply chain disruption | A15 | [35] |
|  | Capacity for supply and demand | Designing generic product | A16 | [42] |
|  |  | Customize generic products depending on the specifications | A17 | [43] |
|  |  | Intelligently utilize delay to meet client needs | A18 | [39] |
|  | Business improvement strategy | Evaluate the degree of risk that key suppliers pose. | A19 | [45] |
|  |  | Detect warning signals of supply chain risk | A20 | [29] |
|  |  | Assess the impact of key suppliers | A21 | [45] |
|  | Crisis strategy | Supply chain contingency strategy reconfiguration. | A22 | [14,18] |
|  |  | Assets reallocation contingency strategy | A23 | [25] |
|  |  | Supply chain disruption recovery contingency strategy | A24 | [38] |
| Supply chain Evolution | Knowledge management | Acquired experience in response to risks | A25 | [51] |
|  |  | Utilization of managing risk experience | A26 | [56] |
|  |  | Earnestly gather social relationship assets | A27 | [56] |
|  | Innovation capacity | Individual innovation potentiality to adapt risks | A28 | [58] |
|  |  | Organizational innovation capability to adapt risks | A29 | [59] |
|  |  | Combined innovation ability to adapt risks | A30 | [60] |
|  | Collaboration among e-commerce supply chain partners | Exchange of knowledge between multinational e-commerce business owners | A31 | [41] |
|  |  | Collaborative planning among multinational e-commerce business owners | A32 | [9] |
|  |  | Interactive collaboration among multinational e-commerce partners | A33 | [65,70] |
|  | Information sharing among partners. | Create a positive business image for fairness | A34 | [61] |
|  |  | Maintaining agreements with multinational e-commerce partners in information sharing. | A35 | [62] |
|  |  | Integrity practices among partners for real-time information exchange | A36 | [63] |

**Section 4: Methodology and Tools**

**Q13. Have you used or are you familiar with the Entropy Weight Method (EWM) for determining criteria weights in supply chain analysis?**

a) Yes

b) No

**Q14. Have you used or are you familiar with the Simple Additive Weighting (SAW) method for ranking factors in supply chain analysis?**

a) Yes

b) No

**Q15. Have you used or are you familiar with Interpretive Structural Modeling (ISM) to evaluate interrelationships among factors in supply chain analysis?**

a) Yes

b) No

**## Data Collection and Analysis**

Table A1: Reliability test

| **Reliability Statistics** | | | | | |
| --- | --- | --- | --- | --- | --- |
| Cronbach's Alpha | | | No. of Items | | |
| .814 | | | 36 | | |
| **Item Statistics** | | | | | |
| Factors | Mean | Std. Deviation | | Corrected Item-Total Correlation | Respondents |
| A1 | 3.6000 | 1.05560 | | .283 | 15 |
| A2 | 3.6667 | .97590 | | .064 | 15 |
| A3 | 2.8667 | .83381 | | .649 | 15 |
| A4 | 3.6000 | .82808 | | .125 | 15 |
| A5 | 3.5333 | .91548 | | .516 | 15 |
| A6 | 2.9333 | .88372 | | .483 | 15 |
| A7 | 4.4000 | .50709 | | .138 | 15 |
| A8 | 3.0000 | .84515 | | .476 | 15 |
| A9 | 4.3333 | .48795 | | -.041 | 15 |
| A10 | 3.3333 | 1.04654 | | .597 | 15 |
| A11 | 4.4667 | .51640 | | .038 | 15 |
| A12 | 4.6000 | .50709 | | .258 | 15 |
| A13 | 4.7333 | .45774 | | .286 | 15 |
| A14 | 4.9333 | .25820 | | .323 | 15 |
| A15 | 3.6000 | .98561 | | .621 | 15 |
| A16 | 4.0000 | .75593 | | .298 | 15 |
| A17 | 3.4667 | .83381 | | -.009 | 15 |
| A18 | 3.7333 | .88372 | | .434 | 15 |
| A19 | 4.0000 | .53452 | | .126 | 15 |
| A20 | 3.8000 | 1.01419 | | .452 | 15 |
| A21 | 3.9333 | .70373 | | .679 | 15 |
| A22 | 3.4000 | 1.05560 | | .330 | 15 |
| A23 | 4.3333 | .61721 | | .547 | 15 |
| A24 | 3.5333 | 1.06010 | | -.034 | 15 |
| A25 | 3.3333 | .89974 | | .344 | 15 |
| A26 | 3.6000 | 1.12122 | | .038 | 15 |
| A27 | 3.6000 | .91026 | | .228 | 15 |
| A28 | 3.4667 | 1.06010 | | .442 | 15 |
| A29 | 3.4000 | .98561 | | .471 | 15 |
| A30 | 4.0667 | .70373 | | .407 | 15 |
| A31 | 4.7333 | .45774 | | .241 | 15 |
| A32 | 4.5333 | .51640 | | .047 | 15 |
| A33 | 4.8000 | .41404 | | .386 | 15 |
| A34 | 4.6000 | .50709 | | -.060 | 15 |
| A35 | 3.8667 | .91548 | | .170 | 15 |
| A36 | 3.5333 | 1.06010 | | .405 | 15 |

Table A2: Consistency Test

|  | Perspectives | | | | |  |
| --- | --- | --- | --- | --- | --- | --- |
| Factors | P1 | P2 | P3 | P4 | P5 | Pi |
| A1 | 1 | 0 | 0 | 1 | 13 | 0.457143 |
| A2 | 12 | 1 | 1 | 1 | 0 | 0.342857 |
| A3 | 2 | 1 | 1 | 0 | 11 | 0.247619 |
| A4 | 12 | 1 | 0 | 1 | 1 | 0.342857 |
| A5 | 1 | 1 | 1 | 12 | 0 | 0.342857 |
| A6 | 2 | 1 | 11 | 1 | 0 | 0.247619 |
| A7 | 0 | 1 | 0 | 14 | 0 | 0.580952 |
| A8 | 1 | 12 | 1 | 1 | 0 | 0.342857 |
| A9 | 0 | 1 | 0 | 14 | 0 | 0.580952 |
| A10 | 1 | 12 | 1 | 1 | 0 | 0.342857 |
| A11 | 1 | 1 | 2 | 11 | 0 | 0.247619 |
| A12 | 0 | 1 | 0 | 14 | 0 | 0.580952 |
| A13 | 12 | 0 | 1 | 1 | 1 | 0.342857 |
| A14 | 0 | 0 | 1 | 0 | 13 | 0.452381 |
| A15 | 1 | 0 | 0 | 12 | 2 | 0.352381 |
| A16 | 1 | 0 | 1 | 2 | 11 | 0.247619 |
| A17 | 13 | 1 | 1 | 0 | 0 | 0.457143 |
| A18 | 0 | 0 | 1 | 1 | 13 | 0.457143 |
| A19 | 0 | 1 | 14 | 0 | 0 | 0.580952 |
| A20 | 1 | 0 | 1 | 13 | 0 | 0.457143 |
| A21 | 1 | 0 | 13 | 1 | 0 | 0.457143 |
| A22 | 1 | 1 | 1 | 12 | 0 | 0.342857 |
| A23 | 1 | 13 | 1 | 0 | 0 | 0.457143 |
| A24 | 0 | 0 | 1 | 14 | 0 | 0.580952 |
| A25 | 1 | 13 | 0 | 1 | 0 | 0.457143 |
| A26 | 0 | 0 | 3 | 12 | 0 | 0.371429 |
| A27 | 0 | 2 | 0 | 13 | 0 | 0.466667 |
| A28 | 12 | 0 | 2 | 0 | 1 | 0.352381 |
| A29 | 0 | 0 | 0 | 1 | 14 | 0.580952 |
| A30 | 1 | 0 | 0 | 13 | 1 | 0.457143 |
| A31 | 0 | 0 | 2 | 13 | 0 | 0.466667 |
| A32 | 1 | 1 | 12 | 1 | 0 | 0.342857 |
| A33 | 1 | 2 | 1 | 11 | 0 | 0.247619 |
| A34 | 1 | 12 | 1 | 1 | 0 | 0.342857 |
| A35 | 1 | 0 | 0 | 0 | 14 | 0.580952 |
| A36 | 0 | 1 | 1 | 13 | 0 | 0.457143 |
| Pj | 0.1521335807 | 0.1484230056 | 0.1410018553 | 0.3821892393 | 0.1762523191 | K= 0.2290 |

**## Evaluation of Interrelationship Among the Factors (ISM Method)**

Table B1: Reachability Matrix (RM)

| **Variables** | **1** | **2** | **3** | **4** | **5** | **6** | **7** | **8** | **9** | **10** | **11** | **12** | **13** | **14** | **15** | **Driving Power** |
| --- | --- | --- | --- | --- | --- | --- | --- | --- | --- | --- | --- | --- | --- | --- | --- | --- |
| 1 | 1 | 0 | 1 | 0 | 0 | 0 | 0 | 0 | 0 | 0 | 0 | 0 | 0 | 1 | 1 | 4 |
| 2 | 0 | 1 | 0 | 0 | 0 | 1 | 0 | 0 | 0 | 0 | 0 | 1 | 0 | 0 | 0 | 3 |
| 3 | 1 | 1 | 1 | 0 | 0 | 0 | 0 | 0 | 1 | 1 | 0 | 1 | 0 | 1 | 1 | 8 |
| 4 | 0 | 0 | 0 | 1 | 0 | 1 | 1 | 0 | 0 | 0 | 0 | 0 | 0 | 0 | 0 | 3 |
| 5 | 0 | 0 | 0 | 0 | 1 | 0 | 0 | 1 | 1 | 1 | 0 | 0 | 0 | 0 | 0 | 4 |
| 6 | 0 | 0 | 0 | 1 | 0 | 1 | 1 | 0 | 0 | 0 | 0 | 0 | 0 | 0 | 0 | 3 |
| 7 | 0 | 0 | 0 | 0 | 0 | 0 | 1 | 0 | 0 | 0 | 0 | 0 | 0 | 0 | 0 | 1 |
| 8 | 0 | 1 | 0 | 0 | 0 | 1 | 0 | 1 | 1 | 1 | 1 | 0 | 0 | 0 | 0 | 6 |
| 9 | 1 | 0 | 0 | 0 | 0 | 0 | 0 | 0 | 1 | 0 | 0 | 0 | 0 | 0 | 0 | 2 |
| 10 | 1 | 0 | 0 | 0 | 0 | 0 | 0 | 0 | 0 | 1 | 0 | 0 | 0 | 0 | 0 | 2 |
| 11 | 0 | 0 | 0 | 0 | 0 | 0 | 1 | 0 | 0 | 0 | 1 | 0 | 0 | 0 | 0 | 2 |
| 12 | 0 | 0 | 0 | 0 | 0 | 0 | 0 | 0 | 0 | 0 | 0 | 1 | 0 | 0 | 0 | 1 |
| 13 | 0 | 0 | 0 | 0 | 0 | 0 | 0 | 0 | 0 | 0 | 0 | 0 | 1 | 0 | 0 | 1 |
| 14 | 0 | 0 | 0 | 0 | 0 | 0 | 0 | 0 | 0 | 0 | 0 | 0 | 0 | 1 | 0 | 1 |
| 15 | 0 | 0 | 0 | 0 | 0 | 0 | 0 | 0 | 0 | 0 | 0 | 0 | 0 | 0 | 1 | 1 |
| Dependence Power | 4 | 3 | 2 | 2 | 1 | 4 | 4 | 2 | 4 | 4 | 2 | 3 | 1 | 3 | 3 |  |

Table B2: Level Partitioning Iterations

| **Elements (Mi)** | **Reachability Set R(Mi)** | **Antecedent Set A(Ni)** | **Intersection Set R(Mi)∩A(Ni)** | **Level** |
| --- | --- | --- | --- | --- |
| 1 | 1, 2, 3, 4, 6, 7, 9, 10, 12, 14, 15 | 1, 3, 5, 8, 9, 10 | 1, 3, 9, 10 |  |
| 2 | 2, 4, 6, 7, 12 | 1, 2, 3, 5, 8, 9, 10 | 2 |  |
| 3 | 1, 2, 3, 4, 6, 7, 9, 10, 12, 14, 15 | 1, 3, 5, 8, 9, 10 | 1, 3, 9, 10 |  |
| 4 | 4, 6, 7 | 1, 2, 3, 4, 5, 6, 8, 9, 10 | 4, 6 |  |
| 5 | 1, 2, 3, 4, 5, 6, 7, 8, 9, 10, 11, 12, 14, 15 | 5 | 5 |  |
| 6 | 4, 6, 7 | 1, 2, 3, 4, 5, 6, 8, 9, 10 | 4, 6 |  |
| 7 | 7 | 1, 2, 3, 4, 5, 6, 7, 8, 9, 10, 11 | 7 | 1 |
| 8 | 1, 2, 3, 4, 6, 7, 8, 9, 10, 11, 12, 14, 15 | 5, 8 | 8 |  |
| 9 | 1, 2, 3, 4, 6, 7, 9, 10, 12, 14, 15 | 1, 3, 5, 8, 9, 10 | 1, 3, 9, 10 |  |
| 10 | 1, 2, 3, 4, 6, 7, 9, 10, 12, 14, 15 | 1, 3, 5, 8, 9, 10 | 1, 3, 9, 10 |  |
| 11 | 7, 11 | 5, 8, 11 | 11 |  |
| 12 | 12 | 1, 2, 3, 5, 8, 9, 10, 12 | 12 | 1 |
| 13 | 13 | 13 | 13 | 1 |
| 14 | 14 | 1, 3, 5, 8, 9, 10, 14 | 14 | 1 |
| 15 | 15 | 1, 3, 5, 8, 9, 10, 15 | 15 | 1 |

Table B3: Level Partitioning Iterations

| **Elements (Mi)** | **Reachability Set R(Mi)** | **Antecedent Set A(Ni)** | **Intersection Set R(Mi)∩A(Ni)** | **Level** |
| --- | --- | --- | --- | --- |
| 1 | 1, 2, 3, 4, 6, 9, 10 | 1, 3, 5, 8, 9, 10 | 1, 3, 9, 10 |  |
| 2 | 2, 4, 6 | 1, 2, 3, 5, 8, 9, 10 | 2 |  |
| 3 | 1, 2, 3, 4, 6, 9, 10 | 1, 3, 5, 8, 9, 10 | 1, 3, 9, 10 |  |
| 4 | 4, 6 | 1, 2, 3, 4, 5, 6, 8, 9, 10 | 4, 6 | 2 |
| 5 | 1, 2, 3, 4, 5, 6, 8, 9, 10, 11 | 5 | 5 |  |
| 6 | 4, 6 | 1, 2, 3, 4, 5, 6, 8, 9, 10 | 4, 6 | 2 |
| 7 |  | 1, 2, 3, 4, 5, 6, 8, 9, 10, 11 |  | 1 |
| 8 | 1, 2, 3, 4, 6, 8, 9, 10, 11 | 5, 8 | 8 |  |
| 9 | 1, 2, 3, 4, 6, 9, 10 | 1, 3, 5, 8, 9, 10 | 1, 3, 9, 10 |  |
| 10 | 1, 2, 3, 4, 6, 9, 10 | 1, 3, 5, 8, 9, 10 | 1, 3, 9, 10 |  |
| 11 | 11 | 5, 8, 11 | 11 | 2 |
| 12 |  | 1, 2, 3, 5, 8, 9, 10 |  | 1 |
| 13 |  |  |  | 1 |
| 14 |  | 1, 3, 5, 8, 9, 10 |  | 1 |
| 15 |  | 1, 3, 5, 8, 9, 10 |  | 1 |

Table B4: Level Partitioning Iterations

| **Elements (Mi)** | **Reachability Set R(Mi)** | **Antecedent Set A(Ni)** | **Intersection Set R(Mi)∩A(Ni)** | **Level** |
| --- | --- | --- | --- | --- |
| 1 | 1, 2, 3, 9, 10 | 1, 3, 5, 8, 9, 10 | 1, 3, 9, 10 |  |
| 2 | 2 | 1, 2, 3, 5, 8, 9, 10 | 2 | 3 |
| 3 | 1, 2, 3, 9, 10 | 1, 3, 5, 8, 9, 10 | 1, 3, 9, 10 |  |
| 4 |  | 1, 2, 3, 5, 8, 9, 10 |  | 2 |
| 5 | 1, 2, 3, 5, 8, 9, 10 | 5 | 5 |  |
| 6 |  | 1, 2, 3, 5, 8, 9, 10 |  | 2 |
| 7 |  | 1, 2, 3, 5, 8, 9, 10 |  | 1 |
| 8 | 1, 2, 3, 8, 9, 10 | 5, 8 | 8 |  |
| 9 | 1, 2, 3, 9, 10 | 1, 3, 5, 8, 9, 10 | 1, 3, 9, 10 |  |
| 10 | 1, 2, 3, 9, 10 | 1, 3, 5, 8, 9, 10 | 1, 3, 9, 10 |  |
| 11 |  | 5, 8 |  | 2 |
| 12 |  | 1, 2, 3, 5, 8, 9, 10 |  | 1 |
| 13 |  |  |  | 1 |
| 14 |  | 1, 3, 5, 8, 9, 10 |  | 1 |
| 15 |  | 1, 3, 5, 8, 9, 10 |  | 1 |

Table B5: Level Partitioning Iterations

| **Elements (Mi)** | **Reachability Set R(Mi)** | **Antecedent Set A(Ni)** | **Intersection Set R(Mi)∩A(Ni)** | **Level** |
| --- | --- | --- | --- | --- |
| 1 | 1, 3, 9, 10 | 1, 3, 5, 8, 9, 10 | 1, 3, 9, 10 | 4 |
| 2 |  | 1, 3, 5, 8, 9, 10 |  | 3 |
| 3 | 1, 3, 9, 10 | 1, 3, 5, 8, 9, 10 | 1, 3, 9, 10 | 4 |
| 4 |  | 1, 3, 5, 8, 9, 10 |  | 2 |
| 5 | 1, 3, 5, 8, 9, 10 | 5 | 5 |  |
| 6 |  | 1, 3, 5, 8, 9, 10 |  | 2 |
| 7 |  | 1, 3, 5, 8, 9, 10 |  | 1 |
| 8 | 1, 3, 8, 9, 10 | 5, 8 | 8 |  |
| 9 | 1, 3, 9, 10 | 1, 3, 5, 8, 9, 10 | 1, 3, 9, 10 | 4 |
| 10 | 1, 3, 9, 10 | 1, 3, 5, 8, 9, 10 | 1, 3, 9, 10 | 4 |
| 11 |  | 5, 8 |  | 2 |
| 12 |  | 1, 3, 5, 8, 9, 10 |  | 1 |
| 13 |  |  |  | 1 |
| 14 |  | 1, 3, 5, 8, 9, 10 |  | 1 |
| 15 |  | 1, 3, 5, 8, 9, 10 |  | 1 |

Table B6: Level Partitioning Iterations

| **Elements (Mi)** | **Reachability Set R(Mi)** | **Antecedent Set A(Ni)** | **Intersection Set R(Mi)∩A(Ni)** | **Level** |
| --- | --- | --- | --- | --- |
| 1 |  | 5, 8 |  | 4 |
| 2 |  | 5, 8 |  | 3 |
| 3 |  | 5, 8 |  | 4 |
| 4 |  | 5, 8 |  | 2 |
| 5 | 5, 8 | 5 | 5 |  |
| 6 |  | 5, 8 |  | 2 |
| 7 |  | 5, 8 |  | 1 |
| 8 | 8 | 5, 8 | 8 | 5 |
| 9 |  | 5, 8 |  | 4 |
| 10 |  | 5, 8 |  | 4 |
| 11 |  | 5, 8 |  | 2 |
| 12 |  | 5, 8 |  | 1 |
| 13 |  |  |  | 1 |
| 14 |  | 5, 8 |  | 1 |
| 15 |  | 5, 8 |  | 1 |

Table B7: Level Partitioning Iterations

| **Elements (Mi)** | **Reachability Set R(Mi)** | **Antecedent Set A(Ni)** | **Intersection Set R(Mi)∩A(Ni)** | **Level** |
| --- | --- | --- | --- | --- |
| 1 |  | 5 |  | 4 |
| 2 |  | 5 |  | 3 |
| 3 |  | 5 |  | 4 |
| 4 |  | 5 |  | 2 |
| 5 | 5 | 5 | 5 | 6 |
| 6 |  | 5 |  | 2 |
| 7 |  | 5 |  | 1 |
| 8 |  | 5 |  | 5 |
| 9 |  | 5 |  | 4 |
| 10 |  | 5 |  | 4 |
| 11 |  | 5 |  | 2 |
| 12 |  | 5 |  | 1 |
| 13 |  |  |  | 1 |
| 14 |  | 5 |  | 1 |
| 15 |  | 5 |  | 1 |

Table B8: Conical Matrix (CM)

| **Variables** | **7** | **12** | **13** | **14** | **15** | **4** | **6** | **11** | **2** | **1** | **3** | **9** | **10** | **8** | **5** | **Driving Power** | **Level** |
| --- | --- | --- | --- | --- | --- | --- | --- | --- | --- | --- | --- | --- | --- | --- | --- | --- | --- |
| 7 | 1 | 0 | 0 | 0 | 0 | 0 | 0 | 0 | 0 | 0 | 0 | 0 | 0 | 0 | 0 | 1 | 1 |
| 12 | 0 | 1 | 0 | 0 | 0 | 0 | 0 | 0 | 0 | 0 | 0 | 0 | 0 | 0 | 0 | 1 | 1 |
| 13 | 0 | 0 | 1 | 0 | 0 | 0 | 0 | 0 | 0 | 0 | 0 | 0 | 0 | 0 | 0 | 1 | 1 |
| 14 | 0 | 0 | 0 | 1 | 0 | 0 | 0 | 0 | 0 | 0 | 0 | 0 | 0 | 0 | 0 | 1 | 1 |
| 15 | 0 | 0 | 0 | 0 | 1 | 0 | 0 | 0 | 0 | 0 | 0 | 0 | 0 | 0 | 0 | 1 | 1 |
| 4 | 1 | 0 | 0 | 0 | 0 | 1 | 1 | 0 | 0 | 0 | 0 | 0 | 0 | 0 | 0 | 3 | 2 |
| 6 | 1 | 0 | 0 | 0 | 0 | 1 | 1 | 0 | 0 | 0 | 0 | 0 | 0 | 0 | 0 | 3 | 2 |
| 11 | 1 | 0 | 0 | 0 | 0 | 0 | 0 | 1 | 0 | 0 | 0 | 0 | 0 | 0 | 0 | 2 | 2 |
| 2 | 1* | 1 | 0 | 0 | 0 | 1* | 1 | 0 | 1 | 0 | 0 | 0 | 0 | 0 | 0 | 5 | 3 |
| 1 | 1* | 1* | 0 | 1 | 1 | 1* | 1* | 0 | 1* | 1 | 1 | 1* | 1* | 0 | 0 | 11 | 4 |
| 3 | 1* | 1 | 0 | 1 | 1 | 1* | 1* | 0 | 1 | 1 | 1 | 1 | 1 | 0 | 0 | 11 | 4 |
| 9 | 1* | 1* | 0 | 1* | 1* | 1* | 1* | 0 | 1* | 1 | 1* | 1 | 1* | 0 | 0 | 11 | 4 |
| 10 | 1* | 1* | 0 | 1* | 1* | 1* | 1* | 0 | 1* | 1 | 1* | 1* | 1 | 0 | 0 | 11 | 4 |
| 8 | 1* | 1* | 0 | 1* | 1* | 1* | 1 | 1 | 1 | 1* | 1* | 1 | 1 | 1 | 0 | 13 | 5 |
| 5 | 1* | 1* | 0 | 1* | 1* | 1* | 1* | 1* | 1* | 1* | 1* | 1 | 1 | 1 | 1 | 14 | 6 |
| Dependence Power | 11 | 8 | 1 | 7 | 7 | 9 | 9 | 3 | 7 | 6 | 6 | 6 | 6 | 2 | 1 |  |  |
| Level | 1 | 1 | 1 | 1 | 1 | 2 | 2 | 2 | 3 | 4 | 4 | 4 | 4 | 5 | 6 |  |  |

**Appendix C: Sensitivity Analysis**

Table C1: Si Values for different set of Criteria weight.

| Factors | For CW Set 1 | For CW Set 2 | For CW Set 3 | For CW Set 4 | For CW Set 5 | For CW Set 6 |
| --- | --- | --- | --- | --- | --- | --- |
| A1 | 0.236 | 0.24 | 0.261 | 0.239 | 0.243 | 0.239 |
| A2 | 0.236 | 0.24 | 0.261 | 0.239 | 0.243 | 0.239 |
| A3 | 0.238 | 0.24 | 0.269 | 0.274 | 0.234 | 0.237 |
| A4 | 0.2 | 0.2 | 0.2 | 0.2 | 0.2 | 0.2 |
| A5 | 0.2 | 0.2 | 0.2 | 0.2 | 0.2 | 0.2 |
| A6 | 0.2 | 0.2 | 0.2 | 0.2 | 0.2 | 0.2 |
| A7 | 0.562 | 0.56 | 0.562 | 0.52 | 0.552 | 0.599 |
| A8 | 0.2 | 0.2 | 0.2 | 0.2 | 0.2 | 0.2 |
| A9 | 0.526 | 0.52 | 0.5 | 0.482 | 0.508 | 0.559 |
| A10 | 0.2 | 0.2 | 0.2 | 0.2 | 0.2 | 0.2 |
| A11 | 0.636 | 0.64 | 0.661 | 0.639 | 0.643 | 0.639 |
| A12 | 0.762 | 0.76 | 0.762 | 0.72 | 0.752 | 0.799 |
| A13 | 0.886 | 0.88 | 0.885 | 0.863 | 0.852 | 0.909 |
| A14 | 1 | 1 | 1 | 1 | 1 | 1 |
| A15 | 0.2 | 0.2 | 0.2 | 0.2 | 0.2 | 0.2 |
| A16 | 0.362 | 0.36 | 0.362 | 0.32 | 0.352 | 0.399 |
| A17 | 0.2 | 0.2 | 0.2 | 0.2 | 0.2 | 0.2 |
| A18 | 0.2 | 0.2 | 0.2 | 0.2 | 0.2 | 0.2 |
| A19 | 0.326 | 0.32 | 0.3 | 0.282 | 0.308 | 0.359 |
| A20 | 0.2 | 0.2 | 0.2 | 0.2 | 0.2 | 0.2 |
| A21 | 0.286 | 0.28 | 0.285 | 0.263 | 0.252 | 0.309 |
| A22 | 0.2 | 0.2 | 0.2 | 0.2 | 0.2 | 0.2 |
| A23 | 0.486 | 0.48 | 0.485 | 0.463 | 0.452 | 0.509 |
| A24 | 0.2 | 0.2 | 0.2 | 0.2 | 0.2 | 0.2 |
| A25 | 0.2 | 0.2 | 0.2 | 0.2 | 0.2 | 0.2 |
| A26 | 0.2 | 0.2 | 0.2 | 0.2 | 0.2 | 0.2 |
| A27 | 0.2 | 0.2 | 0.2 | 0.2 | 0.2 | 0.2 |
| A28 | 0.2 | 0.2 | 0.2 | 0.2 | 0.2 | 0.2 |
| A29 | 0.2 | 0.2 | 0.2 | 0.2 | 0.2 | 0.2 |
| A30 | 0.436 | 0.44 | 0.461 | 0.439 | 0.443 | 0.439 |
| A31 | 0.836 | 0.84 | 0.861 | 0.839 | 0.843 | 0.839 |
| A32 | 0.686 | 0.68 | 0.685 | 0.663 | 0.652 | 0.709 |
| A33 | 0.926 | 0.92 | 0.9 | 0.882 | 0.908 | 0.959 |
| A34 | 0.726 | 0.72 | 0.7 | 0.682 | 0.708 | 0.759 |
| A35 | 0.2 | 0.2 | 0.2 | 0.2 | 0.2 | 0.2 |
| A36 | 0.2 | 0.2 | 0.2 | 0.2 | 0.2 | 0.2 |
